# Research Output of Adult Cardiothoracic Anesthesiology Fellowship Matriculants: A National Cross-Sectional Analysis

**DOI:** 10.7759/cureus.97575

**Published:** 2025-11-23

**Authors:** Sanya Rastogi, Shabaaz M Baig

**Affiliations:** 1 Anesthesiology and Perioperative Medicine, Rutgers University New Jersey Medical School, Newark, USA

**Keywords:** academic medicine, anesthesiology, cardiothoracic anesthesiology, fellowship, graduate medical education, medical education, publication trends, research productivity

## Abstract

Background

Adult cardiothoracic anesthesiology (ACTA) is a highly specialized and increasingly competitive subspecialty within anesthesiology. As research engagement becomes a more prominent component of both residency and fellowship training, scholarly productivity is commonly considered as one of several factors in fellowship selection. However, to our knowledge, no prior national study has evaluated the research output and bibliometric impact of applicants who successfully matched into ACTA fellowships in the United States.

Methods

We conducted a cross-sectional study of ACTA fellows matriculating in the 2024-2025 academic year across programs accredited by the Accreditation Council for Graduate Medical Education (ACGME). Data were obtained from program websites, Doximity (Doximity, Inc., CA, USA), and PubMed/Scopus. Demographic variables included gender, medical degree, and regions of residency and fellowship training. The primary outcome was the total number of peer-reviewed publications per fellow prior to fellowship matriculation. Secondary outcomes included first-author publication count, Scopus h-index, mean Journal Citation Reports (JCR) impact factor of journals published in, and weighted JCR impact factor. Statistical comparisons were made using t-tests and analysis of variance (ANOVA) with post-hoc testing.

Results

Data were available for 211 fellows across 62 programs (80.5% of all positions). Most fellows were male (66.8%) and held M.D. degrees (79.6%). Publication data were found for 201 fellows, of whom 125 (62.2%) had at least one publication. Across the entire cohort, the mean number of total publications was 2.45 ± 4.8 (median = 1), first-author publications was 0.69 ± 2.1 (median = 0), and h-index was 1.53 ± 2.3 (median = 1). Among published fellows, the mean h-index increased to 2.46 ± 2.5, the mean JCR impact factor to 5.23 ± 9.0, and the mean weighted JCR impact factor to 23.4 ± 47.5. No significant gender-based differences were observed in publication counts, h-index, or impact factor metrics. M.D. graduates demonstrated significantly higher total and first-author publications, h-indices, and impact factor values than D.O. graduates (all p < 0.01). Fellows who completed international residency training exhibited the highest overall research productivity, with more total and first-author publications than U.S.-trained peers (all p < 0.05). By fellowship region, fellows training in the West had significantly higher h-indexes, mean JCR impact factors, and weighted impact factors compared with those in other regions (all p < 0.05).

Conclusions

To our knowledge, this is the first national study to quantify both pre-fellowship research productivity and the bibliometric impact of ACTA fellows. While most fellows had modest pre-fellowship publication counts, degree type and training region were significantly associated with scholarly productivity and journal impact metrics. These findings suggest that while research experience may enhance competitiveness, it is not universally required for ACTA fellowship selection. Increased support for research engagement across training environments may help promote equity and academic development within the field.

## Introduction

Adult cardiothoracic anesthesiology (ACTA) is a highly specialized and increasingly competitive subspecialty focused on the perioperative care of patients undergoing complex cardiothoracic procedures. As the field of cardiac anesthesiology has evolved significantly over the past several decades, corresponding with advances in cardiothoracic surgery, interventional cardiology, and perioperative medicine, the role of cardiac anesthesiologists has become more multifaceted [1,2]. As the specialty continues to grow, fellowship programs are not only expanding in clinical scope but also placing greater emphasis on academic development [3]. This trend reflects broader shifts in academic medicine toward evidence-based practice, continuous quality improvement, and innovation in perioperative care. 

Research productivity has become an increasingly analyzed metric across several medical specialties during the fellowship selection process in recent years. Professional organizations such as the Society of Cardiovascular Anesthesiologists (SCA) and the American Society of Anesthesiologists (ASA) advocate for incorporating structured research opportunities into fellowship curricula to advance scientific discovery and professional development [4]. Scholarly activity during fellowship is also considered an important component of academic career advancement and may influence job prospects, faculty appointments, and future research funding [3]. 

Concurrently, bibliometric measures have gained prominence as standardized tools for assessing research impact across specialties. The Scopus h-index quantifies both productivity and citation influence by representing the number h of an author’s publications that have each been cited at least h times, thereby reflecting the cumulative impact of an investigator’s work [5]. The Journal Citation Reports (JCR) impact factor, one of the most widely recognized journal-level metrics, is based on the average number of citations received by articles published in a journal during the preceding two years [6]. These metrics are increasingly used to contextualize the quality and influence of academic output in medical education and career advancement.

Despite the growing importance of research engagement in fellowship training, limited data exist on the academic profiles of applicants to ACTA fellowships, and to our knowledge, no national study has yet characterized the pre-fellowship publication record of matched fellows. Given the increasing emphasis on scholarly activity in anesthesiology residency and fellowship training programs, understanding current norms and trends in publication output may provide valuable insight for prospective applicants.

This study aims to address this gap by analyzing both the research output and bibliometric impact of current ACTA fellows in the U.S. before matching them to their respective fellowship programs. Specifically, we aim to quantify total and first-author publication counts, Scopus h-index, and JCR impact factor metrics to identify associations with gender, medical degree type, and residency or fellowship region. By establishing a benchmark for pre-fellowship research activity, we hope to provide future applicants with a clearer understanding of selection criteria and inform efforts to foster research engagement among trainees.

## Materials and methods

This cross-sectional, observational study was conducted at the New Jersey Medical School, Newark, New Jersey, and aimed to evaluate the pre-fellowship research productivity of ACTA fellows across the United States. All fellowship programs included were accredited by the Accreditation Council for Graduate Medical Education (ACGME). The study focused specifically on fellows anticipated to graduate in the academic year 2024-2025.

Identification of fellowship programs and participants

A comprehensive list of ACGME-accredited ACTA fellowship programs was compiled using the SCA Fellowship Directory [7]. Individual program websites were reviewed to identify the names of fellows anticipated to graduate in 2025. Programs were included if they listed current fellow rosters with identifiable names and training years. Programs were excluded if they did not provide publicly available fellow data. Fellows enrolled in dual ACTA-Critical Care Medicine (CCM) training pathways were excluded from this analysis due to potential confounding influences on research exposure and productivity.

Data collection

Demographic and educational information for each eligible fellow was collected through publicly accessible sources, including fellowship program websites, the online physician network Doximity (Doximity, Inc., CA, USA), and professional social media platforms such as LinkedIn (LinkedIn Corporation, CA, USA). When necessary, multiple sources were cross-referenced to verify accuracy and completeness. The four variables recorded for each fellow were gender, determined based on pronouns, photographs, and biographical descriptions available online, and were classified as male or female (non-binary or unspecified identifications were not encountered); medical degree, M.D., D.O., or Other (international medical degrees, including MBBS, MBChB, and MBBCh); residency program attended prior to fellowship training; and geographic location of residency and fellowship programs as classified by U.S. Census Bureau regions (Northeast, Midwest, South, West, or International for non-U.S. residencies).

Assessment of research productivity

To assess research output, publication data for each fellow were collected from two databases, PubMed and Scopus, using name-based searches that included institutional affiliations and degree credentials to ensure correct attribution. Data collection was restricted to publications indexed on or before June 30, 2023, representing a consistent time frame prior to the final fellowship year. Four metrics were extracted for each individual: total number of peer-reviewed publications listed under the fellow’s name; number of first-author publications, used as a proxy for primary research involvement; Scopus h-index, obtained directly from author profiles on Scopus; and the 2024 JCR impact factor for each journal in which the fellow had published.

In cases of name ambiguity or potential overlap with other authors, publication lists were manually verified using contextual information such as research topics, institutional affiliation, co-author lists, and publication timelines.

Statistical analysis

All statistical analyses were performed using IBM SPSS Statistics for Windows, Version 29 (Released 2022; IBM Corp., Armonk, New York, United States). Descriptive statistics were calculated for demographic variables and reported as counts and percentages. Continuous variables were reported using means, medians, standard deviations (SD), and interquartile ranges (IQR). 

Fellows without any publications or without publications indexed in Scopus were noted to have an h-index of ‘0’. The mean JCR impact factor was calculated using only journals with available 2024 JCR data. 

To account for both publication quantity and journal quality, a weighted publication score was calculated for each fellow by multiplying the number of papers published in each journal by that journal’s JCR impact factor and summing these values across all publications. This composite metric reflects both productivity and the impact of publication. H-index and impact factor data were presented for all fellows and for only those who had at least one publication. 

Group comparisons by gender and degree type were assessed using independent samples t-tests for normally distributed variables. Comparisons across geographic regions were analyzed using one-way analysis of variance (ANOVA). When statistically significant differences were identified, Tukey’s honest significant difference (HSD) test was used for post-hoc pairwise comparisons. A significance threshold of p < 0.05 was used for all statistical tests.

Ethical considerations

All data used in this study were obtained from publicly available sources. No personal or confidential information was accessed or used. As such, the study did not require Institutional Review Board (IRB) approval in accordance with institutional policies regarding research involving publicly accessible data.

## Results

Of the 74 ACGME-accredited ACTA fellowship programs in the U.S., 62 programs (83.8%) provided publicly available fellow data. Around 262 ACTA fellowship positions were filled for the 2024-2025 year, and demographic data were available for 211 fellows (80.53%). Of these, 141 fellows were male (66.8%) and 70 were female (33.2%) (Table 1). Most fellows hold M.D. degrees (79.6%), followed by D.O. degrees (19.0%) and other international degrees (1.4%).

**Table 1:** Gender and degree distribution among ACTA fellows ACTA: adult cardiothoracic anesthesiology

Characteristics	N	Percent
Male	141	66.8
Female	70	33.2
M.D.	168	79.6
D.O.	40	19.0
Other Degrees	3	1.4

Residency program regional data were available for 210 fellows: 33.8% trained in the Northeast, 29.5% in the Midwest, 26.2% in the South, 7.6% in the West, and 2.9% internationally (Table 2). Fellowship program locations showed a similar distribution, with most fellows training in the South (34.1%), followed by the Northeast (30.8%), Midwest (24.2%), and West (10.9%).

**Table 2:** Geographic regions of residency and fellowship training for ACTA fellows ACTA: adult cardiothoracic anesthesiology

Region	Residency Program (N = 210)	Fellowship Program (N = 211)
Northeast	71 (33.8%)	65 (30.8%)
Midwest	62 (29.5%)	51 (24.2%)
South	55 (26.2%)	72 (34.1%)
West	16 (7.6%)	23 (10.9%)
International	6 (2.9%)	-

Publication data were identified for 201 fellows, of whom 125 (62.2%) had at least one peer-reviewed publication. The mean total number of publications per fellow was 2.45 ± 4.8 (median = 1, IQR = 3), with an average of 0.69 ± 2.1 first-author publications (median = 0, IQR = 0) (Table 3). The mean h-index across all fellows was 1.53 ± 2.3 (median = 1, IQR = 2). When restricted to fellows who had published at least one paper (n = 125), the mean h-index increased to 2.46 ± 2.48 (median = 2, IQR = 2). 

**Table 3:** Summary of research output for matched ACTA fellows SD: standard deviation; IQR: interquartile range; ACTA: adult cardiothoracic anesthesiology ^a ^Only fellows with one or more publication(s) included.

Parameters	N	Mean	SD	Median	IQR
Total Publications	201	2.45	4.8	1	3
First-Author Publications	201	0.69	2.1	0	0
H-index	201	1.53	2.3	1	2
H-index (published)^a^	125	2.46	2.48	2	2
Mean JCR Impact Factor	196	3.20	7.5	1.95	3.7
Mean JCR Impact Factor (published)^a^	120	5.23	9.0	2.98	3.4
Weighted Mean JCR Impact Factor	196	14.32	38.8	2.15	10.2
Weighted Mean JCR Impact Factor (published)^a^	120	23.39	47.5	6.15	16.7

Across all fellows, the mean JCR Impact Factor was 3.20 ± 7.5 (median = 1.95, IQR = 3.7), while among published fellows, it rose to 5.23 ± 9.0 (median = 2.98, IQR = 3.4). The mean weighted JCR impact factor, representing the sum of each journal’s impact factor multiplied by the number of fellow-authored publications, was 14.32 ± 38.8 (median = 2.15, IQR = 10.2) across all fellows and 23.39 ± 47.5 (median = 6.15, IQR = 16.7) among published fellows.

No statistically significant gender-based differences were observed in total publications, first-author publications, h-index, mean JCR impact factor, or weighted impact factor (all p > 0.05) (Table 4). However, M.D. fellows exhibited significantly greater research productivity than D.O. fellows. M.D. graduates had a higher mean h-index (p < 0.001), mean JCR impact factor (p = 0.003), and weighted impact factor (p < 0.001). 

**Table 4:** Pre-fellowship publication output by gender, degree type, and region of training Data are presented as 'mean (standard deviation)'. *Independent samples t-test significantly different (p < 0.05). **Tukey’s honest significant difference (HSD) is significantly different than the South (p < 0.05). †Tukey’s HSD is significantly different than the Northeast, Midwest, and South (p < 0.05). JCR: Journal Citation Report

Independent Variable	Groups	Total Publications	First Author Publications	H-Index	Mean JCR Impact Factor	Weighted JCR Impact Factor
Gender						
	Male (n = 135)	2.7 (5.5)	0.8 (2.3)	1.7 (2.5)	2.9 (4.6)	16.7 (45.1)
	Female (n = 66)	1.9 (2.8)	0.5 (1.4)	1.3 (1.7)	3.9 (11.3)	9.4 (20.3)
Degree						
	M.D. (n = 158)	2.8 (5.3)*	0.8 (2.3)*	1.7 (2.5)*	3.6 (8.2)*	17.0 (43.0)*
	D.O. (n = 40)	1.0 (1.6)	0.2 (0.5)	0.7 (1.2)	1.4 (2.0)	3.4 (6.0)
Region of Residency						
	Northeast (n = 69)	2.5 (4.1)	0.6 (1.5)	1.6 (2.6)	3.7 (11.1)	15.7 (36.8)
	Midwest (n = 59)	2.5 (5.3)	0.8 (2.5)	1.6 (2.1)	3.2 (5.9)	12.2 (33.5)
	South (n = 53)	1.5 (2.1)	0.4 (1.0)	1.2 (1.7)	2.2 (2.6)	7.4 (15.3)
	West (n = 14)	3.1 (7.8)	1.0 (3.5)	1.5 (2.6)	4.1 (5.8)	28.6 (75.7)
	International (n = 5)	8.4 (12.0)**	3.6 (4.3)^†^	3.2 (4.4)	3.3 (3.2)	51.9 (102.7)
Region of Fellowship						
	Northeast (n = 63)	2.8 (4.9)	0.7 (2.0)	1.5 (2.2)	2.6 (3.5)	14.2 (35.4)
	Midwest (n = 48)	2.3 (4.7)	0.5 (1.5)	1.5 (2.1)	3.1 (6.3)	12.2 (36.5)
	South (n = 68)	1.7 (3.4)	0.5 (2.0)	1.2 (1.7)	2.2 (2.5)	6.9 (14.4)
	West (n = 22)	4.3 (7.3)	1.5 (3.0)	2.8 (3.8)**	8.3 (18.7)^†^	41.3 (77.0)^†^

Post-hoc analyses revealed that fellows from international residency programs had significantly higher research productivity compared to those from U.S. programs. Specifically, they produced more first-author publications than fellows from the Northeast (p = 0.01), Midwest (p = 0.02), and South (p = 0.01), and more total publications than fellows from the South (p = 0.02) (Table 5). 

**Table 5:** First-author publications by residency region Data were generated using ANOVA with post-hoc Tukey’s honest significant difference (HSD) testing. (I) Groups are represented as rows, and (J) Groups are represented as columns. Data is presented as ‘mean difference (p-value)’.

Region	Northeast	Midwest	South	West	International
Northeast		-0.15 (0.99)	0.18 (0.99)	-0.41 (0.96)	-3.01 (0.01)
Midwest	0.15 (0.99)		0.33 (0.91)	-0.25 (0.99)	-2.85 (0.02)
South	-0.18 (0.99)	-0.33 (0.91)		-0.58 (0.87)	-3.18 (0.01)
West	0.41 (0.96)	0.25 (0.99)	0.58 (0.87)		-2.60 (0.11)
International	3.01 (0.01)	2.84 (0.02)	3.13 (0.01)	2.60 (0.11)	

Among fellowship regions, post-hoc analyses revealed that fellows training in the West had a significantly higher h-index than those in the South (p = 0.02). Western fellows also published in journals with higher impact, reflected by a greater mean JCR impact factor compared with the Northeast, Midwest, and South (p = 0.01, 0.03, and 0.004, respectively), and a higher weighted JCR impact factor than the same regions (p = 0.02, 0.02, and 0.002, respectively) (Table 6).

**Table 6:** Mean JCR impact factor by fellowship region Data were generated using ANOVA with post-hoc Tukey’s honest significant difference (HSD) testing. (I) Groups are represented as rows, and (J) Groups are represented as columns. Data is presented as ‘mean difference (p-value)’. JCR: Journal Citation Report

Region	Northeast	Midwest	South	West
Northeast		-0.46 (0.99)	0.45 (0.99)	-5.71 (0.01)
Midwest	0.46 (0.99)		0.91 (0.92)	-5.25 (0.03)
South	-0.45 (0.99)	-0.91 (0.92)		-6.16 (0.004)
West	5.71 (0.01)	5.25 (0.03)	6.16 (0.004)	

## Discussion

To our knowledge, this study provides the first national analysis of the pre-fellowship research productivity and bibliometric impact of ACTA fellows in the United States. Our findings reveal considerable variability in scholarly output among fellows, with a median of one total publication (IQR = 3), zero first-author publications (IQR = 0), and an h-index of one (IQR = 2) prior to fellowship. This variability in research productivity suggests that while scholarly activity may enhance an applicant’s academic profile, it is not a consistent determinant of fellowship selection, highlighting the multifactorial criteria by which ACTA programs evaluate candidates. These results offer a useful benchmark for prospective applicants and residency program advisors seeking to understand the academic profile of current fellows in this increasingly competitive subspecialty.

Fellowship competitiveness and research productivity 

In recent years, interest in ACTA fellowships has increased, as reflected by resident preferences. According to a 2014 American Board of Anesthesiology survey of CA-1, CA-2, and CA-3 residents, cardiac anesthesiology emerged as the most popular fellowship choice by the CA-2 year (23%), similar to pain medicine (23%) and pediatric anesthesiology (21%) [8]. ACTA fellowships are also one of the most competitive anesthesiology-related fellowships, with 95.6% of positions being filled in the June 2024 SF Match [9]. In comparison, subspecialties such as pain medicine and pediatric anesthesiology had fill rates of 84.5% and 70.7%, respectively, that same year [10,11]. Given this competitiveness, factors influencing fellowship selection have become a topic of interest. National surveys show that fellowship program directors increasingly value meaningful research participation when offering interviews; in fellowship selection, 63% of pain medicine fellowship program directors considered peer-reviewed publications to be of high importance [12]. In that same study, 93% of program directors reported that one to five peer-reviewed publications are appropriate prior to matching. While academic medicine continues to place growing emphasis on research, particularly in subspecialty training, it remains unclear how strongly research productivity impacts fellowship outcomes. 

Despite the growing emphasis on research within academic medicine, we observed that the majority of ACTA fellows had limited publication output before fellowship, highlighting that research productivity may not be a strict prerequisite for fellowship selection. This aligns with prior studies in multiple anesthesiology subspecialties, which have similarly shown that while research engagement is encouraged, it is one of many factors considered in applicant selection [12,13]. For example, a survey of ACTA fellowship directors found that letters of recommendation, in-training exam (ITE) scores, residency program reputation, basic exam scores, United States Medical Licensing Examination (USMLE) scores, and personal statements were all given greater weight than relevant cardiothoracic research when considering applicants for fellowship positions [13]. This is consistent with broader patterns observed in other specialties. Studies of multiple surgical fellowships have found that while research experience enhances applications, program directors often prioritize holistic assessments, including letters of recommendation, interpersonal skills, interview performance, and professionalism and ethics [14-17]. Together, these findings suggest that while research experience can strengthen an application, fellowship selection in ACTA and other subspecialties continues to rely on a comprehensive evaluation of an applicant’s overall clinical performance, character, and fit with the program.

Notably, the inclusion of bibliometric indicators such as the Scopus h-index and JCR impact factor provides a more nuanced understanding of research engagement among fellows. The mean h-index of 1.5 across all fellows (2.5 among those with ≥1 publication) suggests that while most ACTA trainees have initiated scholarly activity, few have produced work that has achieved sustained citation impact. This is consistent with bibliometric benchmarks in early-career anesthesiologists, where the median h-index is estimated to be 1-1.5 [18,19]. The median JCR impact factor of 1.95 further indicates that most fellow-authored publications appeared in moderate-impact journals. However, the broad range of weighted impact factors (IQR = 10.2) suggests that a subset of fellows are publishing in higher-tier journals, potentially reflecting increased mentorship or research infrastructure during their training.

Gender parity in research output

Although only 33.2% of ACTA fellows are female, our analysis found no significant difference in research output between male and female ACTA fellows. This aligns with emerging evidence showing narrowing gender disparities in anesthesiology authorship and academic advancement [20-22]. Recent bibliometric analyses have shown increased female first authorship and editorial representation in major journals, although gaps persist in senior authorship roles [21,22]. A study analyzing two high-impact anesthesiology journals, Anesthesiology and Anesthesia & Analgesia (Lippincott Williams & Wilkins, PA, USA), reported that in 2017, women comprised 30.2% of first authors and 22.6% of last authors [21]. When focusing specifically on cardiac anesthesiology-related articles published between 2002 and 2017, the proportions were lower, with 19.5% female first authorship and 11.6% female last authorship. These findings underscore the importance of sustained mentorship and institutional support to promote gender equity in research productivity and academic leadership.

Degree type and disparities in research opportunities

Fellows with M.D. degrees had significantly more publications and first-author articles than those with D.O. degrees. M.D. graduates also demonstrated significantly higher mean h-indices and weighted JCR impact factors, indicating not only greater quantity but also higher-impact publication output. This disparity may reflect underlying structural differences in research infrastructure, access to mentorship, and emphasis on scholarly activity between allopathic and osteopathic institutions [23]. Previous studies have shown that D.O. students and residents report fewer opportunities to participate in academic research, contributing to underrepresentation in competitive residency, fellowship, and faculty positions [24-26]. Efforts to bridge this gap could include creating dedicated research pathways, expanding access to collaborative networks, and promoting research electives across osteopathic institutions.

International medical graduates and strategic research engagement

Fellows who completed residency training outside the U.S. had the highest average publication counts, particularly in first-author roles. While the sample size was small, this aligns with prior findings that international medical graduates (IMGs) frequently pursue research to strengthen their applications in a competitive U.S. training environment [27]. IMGs have historically relied on research, observerships, and volunteerism to enhance their visibility and competitiveness when faced with systemic barriers such as visa restrictions or limited clinical exposure [28,29]. Supporting this trend, the 2024 NRMP Charting Outcomes™ report found that matched non-U.S. IMGs entering anesthesiology had a mean of 12.0 abstracts, presentations, and publications compared to 9.0 among matched U.S. M.D. seniors [30]. In our analysis, international graduates also demonstrated higher mean h-indices and weighted JCR impact factors than U.S.-trained peers, suggesting that their research not only included more publications but also achieved greater bibliometric influence. These findings highlight the disproportionate emphasis placed on research productivity for IMGs and underscore the need to recognize the structural barriers that contribute to these disparities. 

Regional differences in research output

Significant differences in publication productivity were observed across U.S. residency regions. Fellows from Southern residency programs had lower publication output compared to those from the West and international programs. These findings are consistent with research in other specialties that highlight regional variation in academic culture, research mentorship availability, and access to institutional resources. Fellows training in the Western U.S. demonstrated the highest mean h-index and JCR impact factor, suggesting that institutions in this region may provide greater exposure to high-impact collaborative research environments. Studies in orthopedic surgery residency and fellowship programs have also found significantly lower research output in the Southern region compared to other U.S. regions, particularly the Northeast and West [31,32]. These findings confirm that where a trainee’s residency program is located can correlate with both research quantity and bibliometric quality, with Southern training programs often lagging behind in publication productivity and impact. Addressing these disparities may require cross-institutional initiatives such as shared research platforms, virtual mentorship programs, and national funding opportunities that promote academic engagement across all residency settings.

Limitations

This study has several limitations. First, our reliance on publicly available data may have led to misclassification or underreporting of publication activity, despite cross-referencing multiple databases. Additionally, our publication count does not account for other forms of scholarship not recorded on PubMed or Scopus, such as quality improvement initiatives, non-indexed abstracts or presentations, or book chapters. Because the analysis included only publications indexed in PubMed and Scopus, all journals met basic indexing standards; however, JCR impact factor does not fully capture methodological rigor or scientific quality, and the proliferation of lower-impact or pay-to-publish journals remains a limitation in interpreting bibliometric trends.

## Conclusions

In summary, ACTA fellows in the U.S. demonstrate a wide range of pre-fellowship research productivity, with modest median publication counts and notable differences based on degree type and region of residency training. These findings suggest that while research experience is common among ACTA fellows, it is not universally required, and selection criteria remain multifactorial. Greater support for research engagement across training environments may help broaden opportunities for future applicants and promote continued academic growth within cardiothoracic anesthesiology.
